# Purulent Cutaneous Fistula: As the First Symptom of the Late Aortic Stent-Graft Infection—A Case Report and Review of the Literature

**DOI:** 10.1155/2013/421780

**Published:** 2013-07-30

**Authors:** Damian Ziaja, Grzegorz Biolik, Jerzy Chudek, Krzysztof Ziaja

**Affiliations:** ^1^Department of General and Vascular Surgery, Medical University of Silesia, 40-635 Katowice, Poland; ^2^Department and Unit of Pathophysiology, Medical University of Silesia, 40-635 Katowice, Poland

## Abstract

*Purpose*. Aortic stent-graft infection with fistula formation is a rare complication with high mortality rate when treated surgically by stent-graft removal. We report a case of a patient with aortic stent-graft infection, prosthetic-duodenal, and prosthetic-cutaneous fistulas operated without the removal of an infected prosthesis and ineffectively tailored antibiotic therapy. *Case Report*. A 66-year-old patient with high cardiovascular risk and endovascular stent-graft implantation developed a symptomatic infection of the aortic stent graft 42 months after procedure. It was manifested by iliolumbar muscle abscess and two fistulas: prosthetic cutaneous and prosthetic duodenal. The prosthetic-duodenal fistula was excised and separated from the prosthesis. The perforation within the duodenum was closed in layers. Iliolumbar abscess was removed and drainage was effected .The stent graft was left. The patient received tailored antibiotic therapy. He was readmitted to hospital after 4 weeks with symptoms of infection and leakage of pus discharge in the lumbar area. Despite the antibiotic therapy, the total parenteral nutrition of the patient's clinical status and malnutrition deteriorated and he died of cardiac arrest. *Conclusion*. The presented case confirms that leaving off of the infected stent graft in the patient with severe comorbidity and treated with a tailored antibiotic therapy may not be effective.

## 1. Introduction

The massive gastrointestinal haemorrhage form prosthetic-duodenal fistula is a well-known surgical complication that is rarely observed after endovascular stent-graft implantation [1]. In the case of such sargical complication, the closure of the duodenal fistula and the removal of the infected stent graft and its replacement with silver prosthesis, homograft, homogeneous vein, or extra-anatomic bypass are usually carried out; however, the outcome is usually bad [2]. A series of cases of closing the fistula without removing the stent graft, followed by prolonged antibiotic therapy with good 1-year outcomes, were reported [2, 3]. The small number of these complications prevents the development of evidence-based clinical guidelines on how to manage such a patient. 

We report a case of a patient with two fistulas (prosthetic-duodenal and prosthetic-cutaneous fistulas without massive gastrointestinal bleeding) diagnosed four years after a stent-graft implantation for having symptomatic abdominal aortic aneurysm and severe comorbidities. The first symptoms of stent-graft infection were lumbar pain and purulent cutaneous fistula. 

## 2. Case Report

In 2006, a 66-year-old patient was admitted to our department with symptomatic infrarenal abdominal aortic aneurysm. The patient was qualified for endovascular treatment implantation of stent graft into abdominal aortic aneurysm due to high cardiovascular risk (permanent atrial fibrillation, moderate mitral regurgitation, mild aortic-valve stenosis, and low ejection fraction—25%). The preoperative diagnostics was limited to ultrasound examination and selective aortography taking into account the coexisting chronic kidney disease (eGRF MDRD—43 mL/min/1.73 m^2^). After clopidogrel and salicylic acid premedication (75 mg and 325 mg, resp.), the stent graft—Zenith TRI-FAB— was implanted, without any complication. Within 2 months of procedure, pain complaints related to the aneurysm were subsided. Control Angio-CT performed 6 months after-implantation revealed a complete constriction of the aneurysm sac around the stent graft. The presence of an endoleak was excluded.

**Figure 1:**
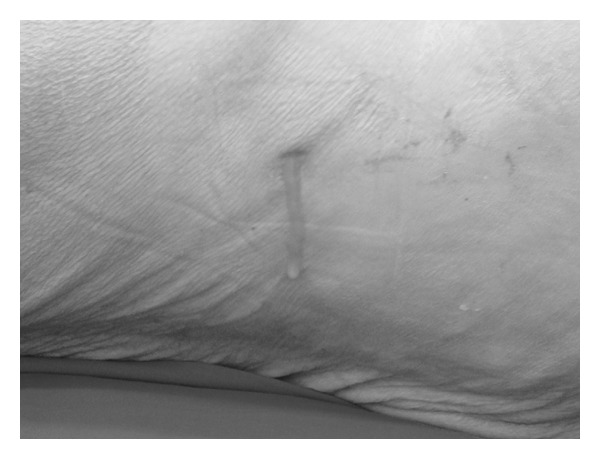
A purulent cutaneous fistula appeared in the lumbar area.

The patient was referred as outpatient with lumbar pain, body mass loss, and escalating weakness after 42 months after-procedure. Laboratory tests revealed increased ESR value and mild leukocytosis (10–12 thousand/mm^3^). Ultrasound examinations of the abdomen and esophagogastroduodenoscopy revealed no pathology. After the next 4 months, a purulent cutaneous fistula appeared in the lumbar area (Figure 1). The patient was referred to the Vascular Surgery Department with the suspicion of enterocutaneous fistula.

**Figure 2:**
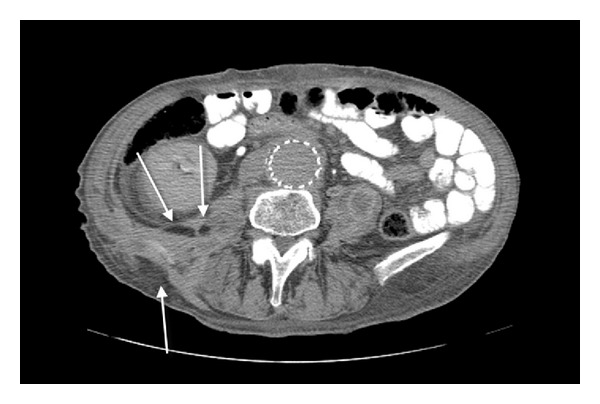
A stent-graft infection with abscess within the psoas muscle and skin fistula.

**Figure 3:**
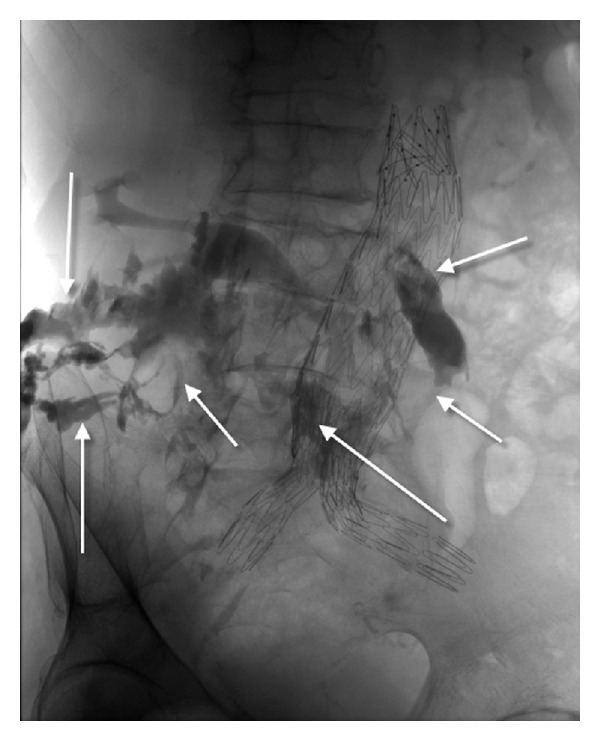
Fistulography: multicanal fistula within the lumbosacral area with contrast accumulation in the small bowel.

After admission, the patient underwent a laboratory and imaging examination. The fistula swab showed the growth of *Streptococcus pneumoniae* strain resistant to penicillin but sensitive to tetracycline and clindamycin. Antibiotic therapy with intravenous clindamycin and oral neomycin was started. Angio-CT revealed stent-graft infection with abscess within the psoas muscle and skin fistula (Figure 2). Fistulography showed multicanal fistula within the lumbosacral area with contrast accumulation in the small bowel (Figure 3). Endoscopy was not performed. 

**Figure 4:**
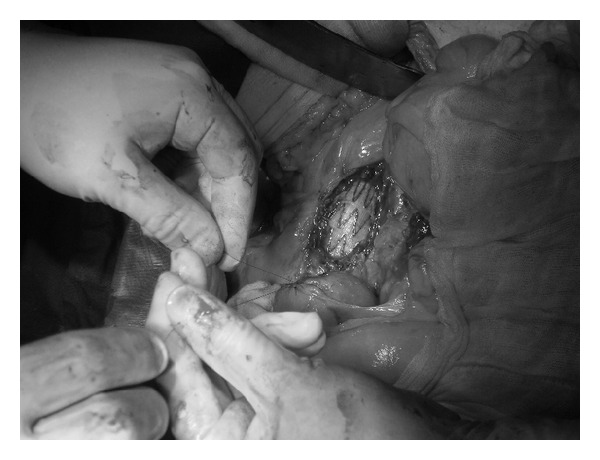
Closure of duodenal perforation.

The patient underwent scheduled surgical treatment, during which a second fistula was revealed, located between the trunk of the prosthesis and the third part of duodenum. The stent graft appeared not to be defected. The prosthetic duodenal fistula was separated from the stent graft and excised, whereas the perforation within the duodenum was closed in layers (Figure 4). The area between the stent graft and duodenum was separated by a part of the omentum. The abscess of the iliolumbar muscle, as well as prosthetic-skin fistula, was isolated from the stent graft with another part of the omentum, cut, and drained. Because of the overall clinical condition of the patient, the infected stent graft was not removed. After surgery, the antibiotic therapy was changed to ciprofloxacin, according to the bacterial strain's sensitivity, and the oral administration of neomycin was continued. On the 10th day after the surgery, the purulent matter of the abscess was evacuated by the drain and granulating fistula. After seven following days of tailored antibiotic therapy with ciprofloxacin, the clinical condition of the patient improved significantly, pain subsided, and the fistula healed over. The control ultrasound examinations of the abdominal cavity did not reveal any pathology around the stent graft. The patient was discharged from the hospital in a good clinical condition and orally nourished. The general practitioner continued the antibiotic therapy and low-molecular-weight heparin as VTE prophylaxis.

**Figure 5:**
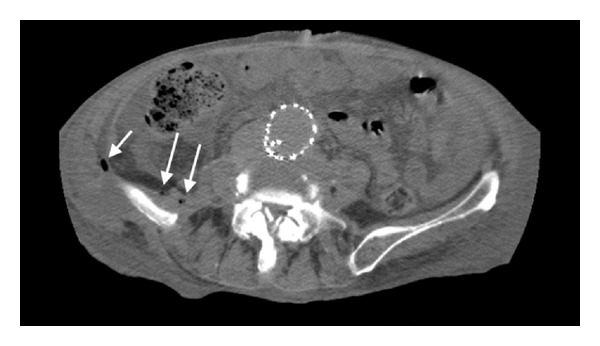
Abscess formation within the retroperitoneal space.

After four weeks, the patient was readmitted to the Vascular Surgery Department with symptoms of infection and leakage of pus discharge in the lumbar area. Bacterial culture showed the growth of *Staphylococcus epidermidis MSSE *strain, sensitive to fluoroquinolones, oxacillin, cephalosporins, and penicillin with **β*-lactamase* inhibitor. Control Angio-CT revealed abscess formation within the retroperitoneal space, without infiltration surrounding the stent graft (Figure 5). Despite the antibiotic therapy and total parenteral nutrition provided, the patient's clinical and nutritional statuses deteriorated. He died of cardiac arrest on the 11th day of hospitalisation.

## 3. Discussion

The frequency of abdominal aorta stent-graft infection is estimated at 0.5–0.7%, and it is lower than that of the infection complications after surgical procedure that ranges from 0.6% to the 3.0% [4, 5]. Many causes may lead to graft infection and formation of fistula, such as local infection, inflammatory aneurysm, mycotic aneurysm, nosocromial infection, or related to EVAR like endoleak, graft migration, kinking, endotension and endoleak embolisation. Typical symptom of graft infection and fistula formation are gastrointestinal bleeding, pulsatile abdominal mass, and pain is present in only 25% of the patients. In the majority of cases, back pain or fever may be the main and only presenting symptom. To the best of our knowledge, it is the first report of simultaneous cutaneous fistula formation in the course of aortic stent-graft infection.

As these complications are rare, no therapeutic algorithm of the stent-graft infection has been developed. In patients treated surgically with the removal of infected stent graft, the mortality rate is extremely high, regardless of the applied reconstruction method (anatomic transplant versus extra-anatomic transplant) the material used (silver prosthesis, homograft, and homogeneous vein), and the patient clinical conditions [6–8]. Bergqvist et al. reported 16 cases of surgically treated patients with stent-graft infection and aortoenteric fistula. In all cases, the main cause of infection and fistulation defect in the stent graft or its function was recognised [9]. Saratzis et al. reported five cases of stent-graft infection treated surgically with a mortality rate of about 40%. Contrary to Bergqvist et al.'s findings, no defect in the explanted grafts was found [10].

Few case reports suggest the restriction of the therapy to prolonged tailored-antibiotic therapy [4, 11, 12]. Even the blood cultures were found to be negative in all the cases. In 2005, Hart et al. reported a series of 15 cases treated with partial removal of the infected stent graft with a mortality rate of 27% [13].

The decision whether to leave the infected stent graft or to remove it surgically should include the comorbidity and anticipated time of patient survival. The majority of patients treated invasively died within a short postoperative period, usually due to cardiac causes, sepsis, or aortic rupture.

## 4. Conclusion

The presented case confirms that the leaving of the infected stent graft in the patient with severe comorbidity, regardless of tailored antibiotic therapy, may not be an effective therapy.
